# One-stage versus two-stage bilateral implantable collamer lens implantation: a comparison of efficacy and safety

**DOI:** 10.1038/s41598-024-54101-0

**Published:** 2024-03-07

**Authors:** Anna Lorger, Nikolaus Luft, Wolfgang J. Mayer, Siegfried G. Priglinger, Martin Dirisamer

**Affiliations:** 1 Department of Ophthalmology, Ludwig-Maximilians-University Munich, Mathildenstrasse 8, 80336 Munich, Germany; 2 SMILE Eyes Linz, Linz, Austria

**Keywords:** Outcomes research, Anatomy

## Abstract

Implantable collamer lens implantation (ICL) represents a safe and effective treatment for myopia and myopic astigmatism. To compare the outcomes of a bilateral one-stage same day approach to a two-stage approach, the databases of the University Eye Hospital Munich, Ludwig Maximilians-University and Smile Eyes Linz, Austria were screened for eyes that had undergone ICL implantation. Two-stage surgery was performed at an interval of 1 day (17 patients), 2 days (19 patients) and 1 week (2 patients). Variables analyzed were preoperative, 1-day and last follow-up uncorrected distance (UDVA) and corrected distance visual acuity (CDVA), manifest refraction, refractive spherical equivalent (SEQ), astigmatism, age, endothelial cell count (ECD), intraocular pressure (IOP) and ICL vaulting. In total, 178 eyes (100 eyes one-stage, 78 eyes two-stage) of 89 patients were included in this study. Mean follow-up was 1.1 ± 0.8 and 1.3 ± 0.5 years. Mean preoperative SEQ was − 7.9 ± 2.6 diopters (D) in the one-stage and − 8.0 ± 1.7 D in the two-stage group (p = 0.63) and improved to 0.00 ± 0.40 and − 0.20 ± 0.40 D at end of follow-up, showing slightly better stability in the one-stage group (p = 0.004). There was no difference in the efficacy (1.1 vs. 1.2, p = 0.06) and the safety index (1.2 vs. 1.2, p = 0.60) between the two groups. No eye (0%) in either group lost 2 lines or more of UDVA (p > 0.99). Refraction within ± 0.50 D and ± 1.00 D around target was achieved comparably often (89 vs. 86%, p = 0.65; 99 vs. 99%, p > 0.99). Endothelial cell loss was slightly higher in the two-stage group (1.3 vs. 4.3%). Vaulting at the final follow up was higher in the one-stage group (373.8 ± 205.4 µm vs. 260.3 ± 153.5 µm, p = 0.00007). There were no serious intraoperative complications in either group. In conclusion, this study demonstrates that both the one- and two-stage approaches are equally effective, predictable and safe. Regarding endothelial cell loss, vaulting and SEQ stability, the one-stage group showed slightly better outcomes, but these results are clinically questionable because they are so small. Larger studies are needed to quantitatively evaluate a potential benefit.

## Introduction

Patients with myopia or severe myopic astigmatism often desire to undergo surgical correction of their refractive errors. Popular refractive corneal procedures such as laser-assisted in situ keratomileusis (LASIK), photorefractive keratectomy (PRK) and small-incision lenticule extraction (SMILE) are well-established options but can be limited by corneal parameters or the sheer magnitude of correction in high myopia, hyperopia or astigmatism.

In those patients, implantable collamer lens (ICL, STAAR Surgical, Monrovia, CA, USA), for the first time approved by the FDA in 2005, represents a safe, reversible alternative with excellent results regarding safety and efficacy^1^. ICL is often used in complex eyes, e.g. due to underlying corneal ectatic disease, where keratorefractive surgery is not possible, or in extreme refractive errors. Currently, ICL correction is available in up to -18 diopters for myopia, up to + 12 diopters for hyperopia and up to + 6 diopters for astigmatism^2^.

As ICL is usually implanted in young patients, safety is of utmost importance, with endophthalmitis being the most severe side effect. On the other hand, the high refractive errors, mostly present in both eyes, require timely bilateral intervention. Therefore, aspects of safety, suggesting a two-stage approach on two different days, must be balanced with the problem of postoperative anisometropia, cost-efficacy and the convenience of the patient, suggesting a one-stage approach.

Unfortunately, no comparative studies on one-stage vs. two-stage bilateral ICL implantation are available. Therefore, the question arises if one-stage surgery on the same day or two-stage surgery is safer for the patient. To overcome this current research gap, hypothesis of this study was to show non-inferiority of a same-day one-stage procedure compared to a two-stage procedure concerning safety and efficacy.

## Results

In this study, 178 eyes of 89 patients (66 female, 23 male) who underwent ICL implantation in the period 2013–2020 were retrospectively analyzed. Of these, 100 eyes of 50 patients (32 female, 18 male) were operated as a one-stage procedure, i.e. both eyes were operated on one day directly after each other; and 78 eyes of 39 patients (34 female, 5 male) were operated as a two-stage procedure with an interval of 1 day (17 patients), 2 days (19 patients) or 1 week (2 patients).

The mean age of the patients operated as one-stage procedure was 33.4 ± 6.5 years (range: 21.7 ± 49.0), that of the patients operated as two-stage procedure 33.1 ± 6.8 years (range: 21.5 to 46.3; p = 0.80). Mean follow-up was 1.1 ± 0.8 years in the one-stage group and 1.3 ± 0.5 years in the two-stage group.

Table 1 shows the preoperative characteristics and their P values of both groups. There was no difference in either parameter between both groups except for mean follow-up, which however should be clinically negligible.

**Table 1:** Baseline characteristics of the two treatment groups.

Parameter	One-stage		Two-stage		P-value
	Mean ± SD	Range	Mean ± SD	Range	
Gender	32 w, 18 m		34w, 5 m		
Age (years)	33.4 ± 6.5	21.7, 49.0	33.1 ± 6.8	21.5, 46.3	0.798
CDVA (logMAR)	−0.1 ± 0.2	0.6, 1.5	−0.1 ± 0.1	1, 1.6	0.064
Sphere (D)	− 7.2 ± 2.6	− 13.75, − 2.25	− 7.3 ± 1.7	− 11.25, − 3.8	0.116
Cylinder (D)	− 1.4 ± 1.2	− 5.0, 0.0	− 1.4 ± 1.1	− 4, 0.0	0.215
Axis	94.7 ± 68.0	0.0, 180	102.3 ± 70.8	1, 180.0	0.368
SEQ (D)	− 7.9 ± 2.6	− 14.5, − 3.1	− 8.0 ± 1.7	− 11.5, − 3.9	0.629
ECD (cells/mm^2^)	2727.1 ± 232.5	2092, 3231	2700.3 ± 218.5	2320, 3089	0.447
IOP (mmHg)	14.8 ± 3.4	9.0, 28.0	15.1 ± 2.8	10, 22.0	0.570

### Efficacy

**Figure 1:**
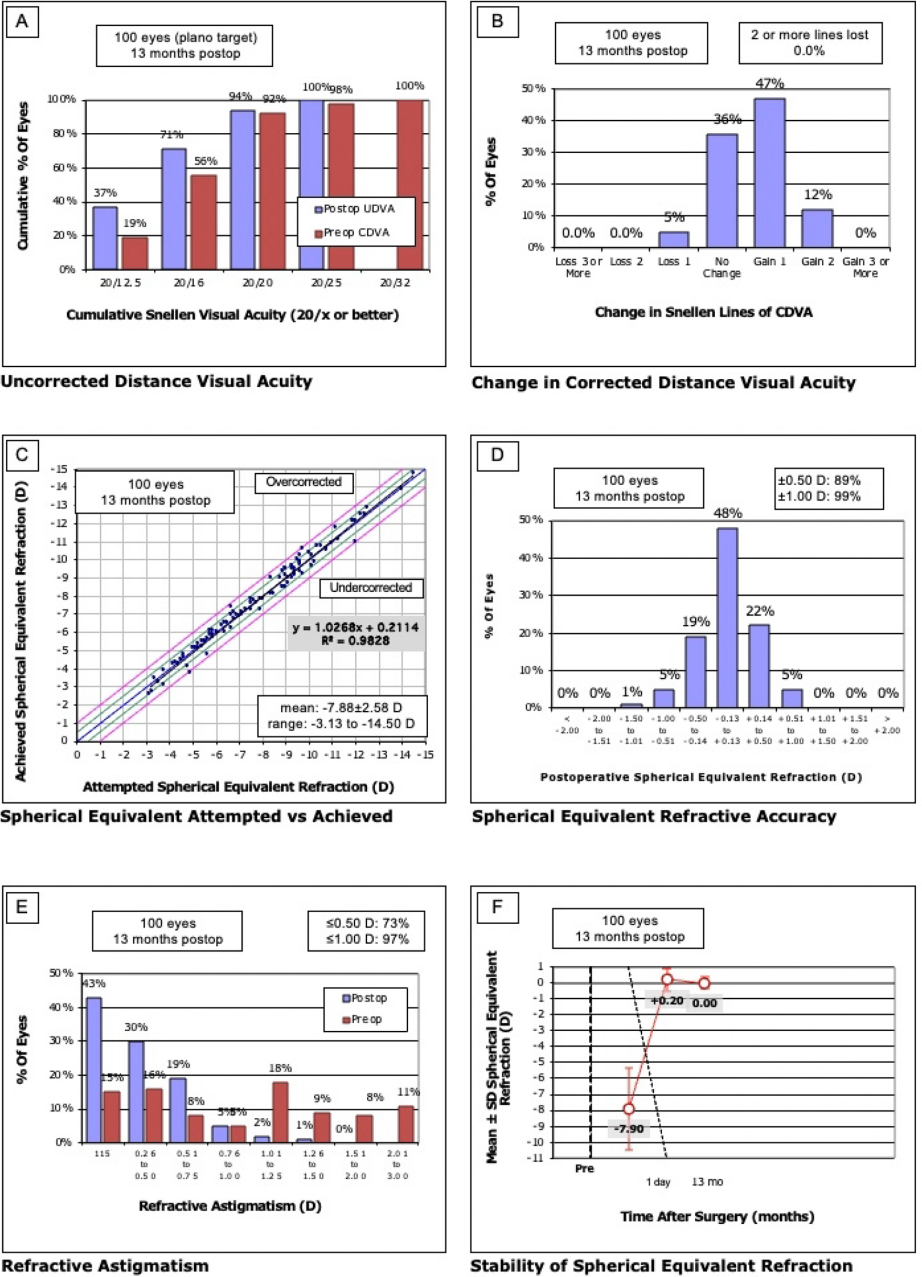
One-stage group. (**A**) Distribution of UDVA postoperatively compared to CDVA preoperatively. All eyes in both groups achieved a UDVA of 20/25 or better, and 94% in the one-stage group achieved 20/20 or better, in the two-stage group 95%. The efficacy indices were comparable one day postoperative (one-stage 1.0 ± 0.3 vs. two-stage 1.0 ± 0.2, p = 0.32) and at the last follow up (one-stage 1.1 ± 0.2 vs. two-stage 1.2 ± 0.2, p = 0.06. (**B**) Change in lines of CDVA and UDVA. No eye in both groups lost 2 or more lines. In the one-stage group 5 eyes (5%) lost one line, in the two-stage group 6 eyes (8%), p = 0.54. The safety index at the last follow up was not significant (one-stage 1.2 ± 0.2 vs. two-stage 1.2 ± 0.2, p = 0.6). (**C**) Attempted vs. achieved spherical equivalent refraction. By definition of spherical equivalent beyond 1.0 D from target refraction, no eye in either group was under- or overcorrected. (**D**) Distribution of manifest spherical equivalent refraction after retreatment. The number of eyes within 0.5 and 1.0 D from target refraction increased was 89% and 99% in the one-stage group, 86% and 99% in the two-stage group. There was a significant difference in the final target accuracy (p = 0.004). (**E**) Distribution of refractive astigmatism before and after retreatment. After one-stage ICL implantation, the number of eyes with astigmatism ≤ 0.5 and ≤ 1.0 D increased from 31 to 73%, and from to 44% to 97%. After two-stage ICL implantation, the number of eyes with astigmatism ≤ 0.5 and ≤ 1.0 D increased from 20 to 67%, and from to 36% to 96%. There was no significant difference in the final postoperative astigmatism (p = 0.09). (**F**) Refractive stability after enhancement. In both groups, MRSE was comparable one day postoperative (one-stage 0.20 ± 0.70 D vs. two-stage 0.20 ± 0.40 D, p = 0.48).

**Figure 2:**
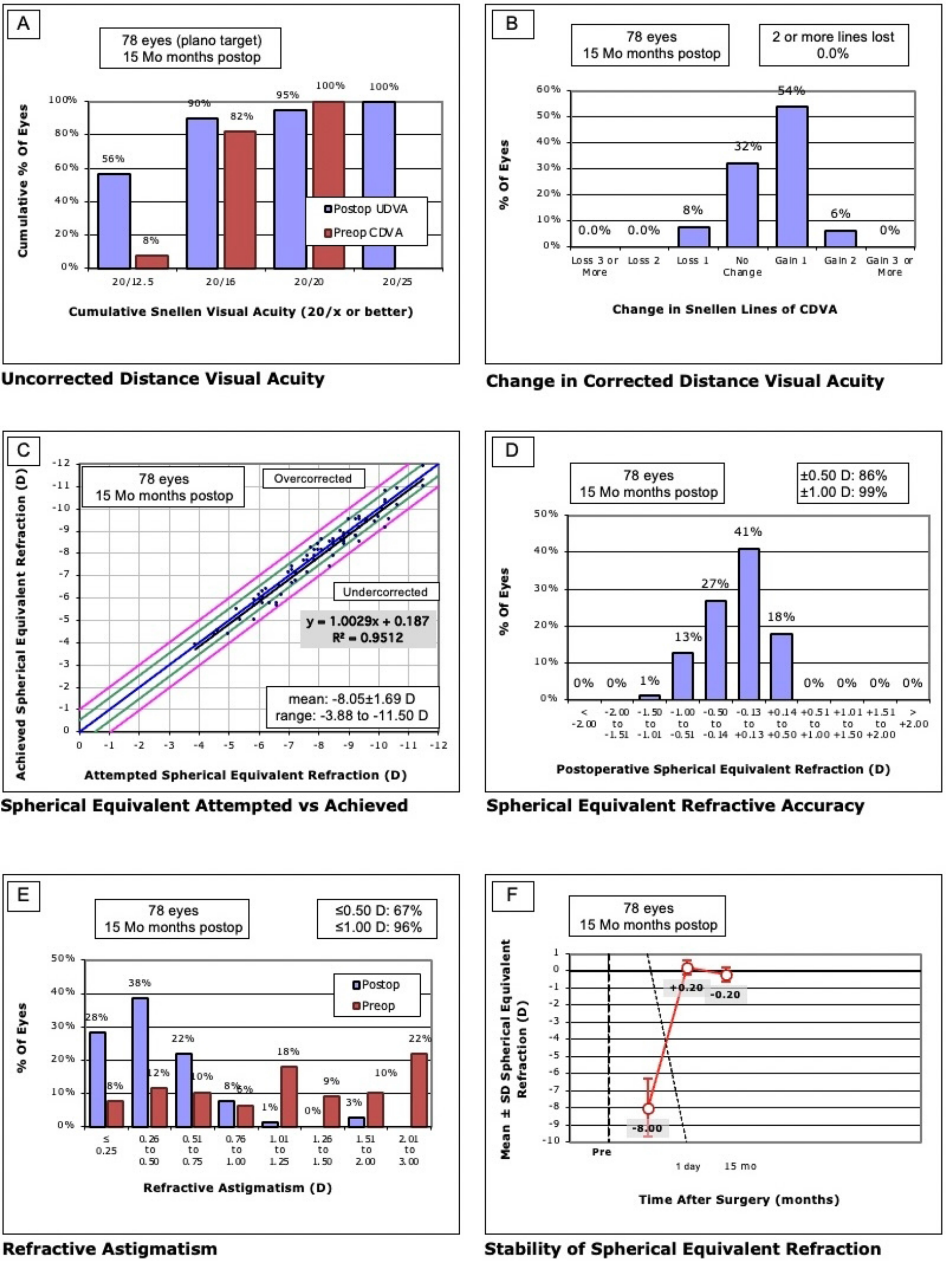
Two-stage group. (**A**) Distribution of UDVA postoperatively compared to CDVA preoperatively. All eyes in both groups achieved a UDVA of 20/25 or better, and 94% in the one-stage group achieved 20/20 or better, in the two-stage group 95%. The efficacy indices were comparable one day postoperative (one-stage 1.0 ± 0.3 vs. two-stage 1.0 ± 0.2, p = 0.32) and at the last follow up (one-stage 1.1 ± 0.2 vs. two-stage 1.2 ± 0.2, p = 0.06. (**B**) Change in lines of CDVA and UDVA. No eye in both groups lost 2 or more lines. In the one-stage group 5 eyes (5%) lost one line, in the two-stage group 6 eyes (8%), p = 0.54. The safety index at the last follow up was not significant (one-stage 1.2 ± 0.2 vs. two-stage 1.2 ± 0.2, p = 0.6). (**C**) Attempted vs. achieved spherical equivalent refraction. By definition of spherical equivalent beyond 1.0 D from target refraction, no eye in either group was under- or overcorrected. (**D**) Distribution of manifest spherical equivalent refraction after retreatment. The number of eyes within 0.5 and 1.0 D from target refraction increased was 89% and 99% in the one-stage group, 86% and 99% in the two-stage group. There was a significant difference in the final target accuracy (p = 0.004). (**E**) Distribution of refractive astigmatism before and after retreatment. After one-stage ICL implantation, the number of eyes with astigmatism ≤ 0.5 and ≤ 1.0 D increased from 31 to 73%, and from to 44% to 97%. After two-stage ICL implantation, the number of eyes with astigmatism ≤ 0.5 and ≤ 1.0 D increased from 20 to 67%, and from to 36% to 96%. There was no significant difference in the final postoperative astigmatism (p = 0.09). (**F**) Refractive stability after enhancement. In both groups, MRSE was comparable one day postoperative (one-stage 0.20 ± 0.70 D vs. two-stage 0.20 ± 0.40 D, p = 0.48).

The cumulative percentage of preoperative CDVA and postoperative UDVA for each group is shown in Figs. 1A and 2A. The cumulative UDVA ≥ 20/25 was 100% in both groups (p > 0.99), ≥ 20/20 in 94% in the one-stage group and ≥ 20/20 in 95% in the two-stage group (p > 0.99), ≥ 20/16 in 71% in the one-stage and ≥ 20/16 in 90% in the two-stage group (p = 0.002). Cumulative visual acuity ≥ 20/12.5 was 37% in the one-stage operated group and 56% in the two-stage operated group (p = 0.02).

The efficacy index was not statistically significantly different between the two groups at day 1 postoperatively (one-stage 1.0 ± 0.3 vs. two-stage 1.0 ± 0.2, p = 0.32) and at the last control examination (one-stage 1.1 ± 0.2 vs. two-stage 1.2 ± 0.2, p = 0.06).

### Safety

As seen in Figs. 1B and 2B, no eye lost 2 or more lines in both groups (0%, p > 0.99). In the one-stage group 5 eyes (5%) lost 1 line, in the two-stage group 6 eyes (8%; p = 0.54). The safety index of both groups at the final follow-up (one-stage 1.2 ± 0.2 vs. two-stage 1.2 ± 0.2) was not statistically significantly different (p = 0.60).

### Predictability

The expected versus achieved SEQ correction of the respective groups is shown in Fig. 1, C + D and in Fig. 2, C + D. In the one-stage group, 89 eyes (89%) had achieved target within ± 0.50 D of expected refraction, in the two-stage group 67 eyes (86%). This is not statistically significantly different (p = 0.65). Target within ± 1.0 D of expected refraction was achieved equally often in both groups with 99% (one-stage 99 eyes vs. two-stage 77 eyes, p > 0.99). Comparing both groups preoperatively and 1 day postoperatively regarding SEQ, no significant difference was found at both observation periods (p = 0.63 preoperatively, p = 0.48 1 day postoperatively). End of follow-up shows a statistically significant result in favor of the one-stage group (p = 0.004) with however only a small difference (0.00 ± 0.40 vs. − 0.20 ± 0.40 D).

The development of refractive astigmatism of the two groups is shown in Figs. 1E and 2E. In the one-stage group, 73 (73%) had a postoperative astigmatism of ≤ 0.5 D, in the two-stage group 52 eyes (67%; p = 0.41). Both groups had a comparable postoperative astigmatism percentage ≤ 1.0 D (one-stage 97 eyes (97%) vs. two-stage 75 eyes (96%), p > 0.99). When comparing both groups regarding refractive astigmatism, there was a statistically non-significant result at all observation time points (preoperative p = 0.21, 1-day postoperative p = 0.27, final follow-up p = 0.09).

### Stability

Figures 1F and 2F show the stability of the spherical equivalent (SEQ) of the two groups.

A similar spherical equivalent 1 day postoperatively was observed in both groups (one-stage 0.20 ± 0.70 D vs. two-stage 0.20 ± 0.40 D, p = 0.48). At final follow-up of 1 year, the spherical equivalent was 0.00 ± 0.40 D in the one-stage group and -0.20 ± 0.40 D in the two-stage group. This shows a statistically significant difference between the groups (p = 0.004) suggesting slight myopic regression in the two-stage group.

### Vaulting

When comparing the vaulting of both groups, there was a statistically significant difference in the 180-degree meridian Scheimpflug section. In the one-stage group, the 180-degree meridian vaulting was 373.8 ± 205.4 µm in the one-stage group, 260.3 ± 153.5 µm in the two-stage group (p = 0.00007).

### Endothelial cell count

Endothelial cell count decreased in the one-stage group from 2727.1 ± 232.5 cells/mm^2^ preoperatively to 2691.6 ± 287.8 cells/mm^2^ at the last follow-up (1.3%). In the two-stage group, there was a loss of endothelial cell number from 2700.3 ± 218.5 cells/mm^2^ preoperatively to 2584.1 ± 277.3 cells/mm^2^ at the final follow-up. This represents a percentage of 4.3%.

The comparison of both groups preoperatively shows a statistically non-significant result with p = 0.45. One day postoperatively (p = 0.019) and at the final control (p = 0.016) both results are statistically significantly different with more endothelial cell loss in the two-stage group.

### Intraocular eye pressure

The comparison of mean intraocular pressure of both groups showed no differences both preoperatively (p = 0.57), 1 day postoperatively (p = 0.33) and at the final follow-up (p = 0.61).

The results are shown in Table 2.

**Table 2:** Perioperative development of intraocular pressure.

Mean intraocular pressure (IOD)	One-stage		Two-stage		P-value
	Mean + SD	Range	Mean + SD	Range	
Preoperative	14.8 ± 3.4	9.0, 28.0	15.1 ± 2.8	10.0, 22.0	0.569
1 day postoperative	14.8 ± 3.8	8.0, 27.0	14.3 ± 3.1	8.0, 25.0	0.332
Final follow up	15.1 ± 3.2	8.0, 23.0	15.2 ± 3.0	9.0, 20.0	0.610

### Perioperative and postoperative complications

Table 3 shows the complications of both groups. There were no severe intraoperative or postoperative complications in either group. In both groups, halos were the most frequently perceived postoperative symptoms (one-stage 4% vs. two-stage 5.1%, p = 0.73). In either group, one patient had a toric ICL-rotation (one stage 1% vs. two-stage 1.3%, p > 0.99) but with no need for repeat surgery. In the one-stage group because of high vault and in the two-stage group because of a rest-astigmatism of 1.25 D. In either group, a similar percentage of patients developed macular edema postoperatively (one-stage 4% vs. two-stage 3.8%, p > 0.99) which was treated with topical nonsteroidal anti-inflammatory eye drops in all cases. Regarding postoperative IOP spikes, the percentage of eyes with a transient mild increase > 21 mmHg on day one postoperatively was 6 eyes (6%) in the one-stage and 2 eyes (2.6%) in the two-stage group (p = 0.47). At the last follow up, 2 eyes (2%) in the one-stage group and no eye (0%) in the two-stage group had an increased IOP > 21 mmHg (p = 0.50). This had no clinical relevance in all cases and was treated with bimatoprost and brimonidin combination therapy for maximum 4 weeks. None of the patients had an increased risk of glaucoma.

**Table 3:** Adverse events and complications of the two treatment groups.

	One-stage	Two-stage	P-value
Injury of neighboring structures	0 (0%)	0 (0%)	> 0.99
Night-vision-symptoms			
Glare	0 (0%)	0 (0%)	> 0.99
Halos	4 (4%)	4 (5.1%)	0.73
Starburst	0 (0%)	1 (1%)	0.44
Abnormal ICL-position			
ICL-dislocation	0 (0%)	0 (0%)	> 0.99
ICL-rotation	1 (1%)	1 (1.3%)	> 0.99
ICL-inversion	0 (0%)	0 (0%)	> 0.99
Endothelial cell number loss cells/mm^2^	1.3 %	4.3 %	0.016
Increased intraocular pressure	2 (4%)	1 (1.3%)	> 0.99
Endophthalmitis	0 (0%)	0 (0%)	> 0.99
Cataract	0 (0%)	0 (0%)	> 0.99
Macular edema	4 (4%)	3 (3.8%)	> 0.99

## Discussion

The current study is the first to systematically analyze the efficacy, safety, predictability, and stability of a one- versus two-stage approach to ICL implantation using a representative cohort and illustrating it with standard refractive surgery graphs. We found that both approaches produce similar results with only clinically negligible differences, slightly favoring a one-stage approach concerning endothelial cell loss, vaulting and SEQ stability.

There are already some studies which investigated whether one-stage or two-stage cataract surgery is preferable. Li et al.^3^ reported that one-stage cataract surgery, i.e. both eyes in one day, is a safe and effective option for cataract. Such a procedure is associated with faster vision rehabilitation as well as a significant cost reduction without additional risk^3^. Lacy et al.^4^ investigated whether the rate of postoperative endophthalmitis is higher with one-stage or two-stage cataract surgery. Ultimately, no significant difference was found^4^.

Over the entire follow-up, ICL implantation as one-stage and two-stage procedure was associated with good efficacy and safety which can be compared with other studies^5,6^. Regarding the stability of the spherical equivalent, the one-stage group performed slightly better at the final follow-up with 0.00 ± 0.40 D compared with the two-stage group with − 0.20 ± 0.40 D, but this is of no clinical significance because of the low value.

Efficacy is supported by the fact that 94% of eyes in the one-stage group and 95% in the two-stage group exhibited an UDVA ≥ 20/20 and the efficacy index at the last follow up was statistically significantly not different (one-stage 1.1 ± 0.2 vs. two-stage 1.2 ± 0.2). These results are well with the results in the study from Abdelmassih et al.^7^ with a follow up of two years or from Kamiya et al.^8^. In our study the efficacy index was slightly better, but the longer follow-up of 8 years in the cited studies might be responsible for this difference.

Regarding safety, there were no severe intraoperative complications in both groups. We observed a comparable high safety index in both groups (one-stage 1.2 ± 0.2 vs. two-stage 1.2 ± 0.2), similar to that reported by Kamiya et al.^8^ (1.18 ± 0.24). No eye of either group lost 2 or more lines. However, in the one-stage group 12 eyes (12%) gain 2 lines, in the two-stage group 4 eyes (6%). This is similar to the results of Shimizu et al.^9^, where 8% gain 2 lines of CDVA. The most important parameter to note is that no endophthalmitis, which is the most severe complication, occurred in any of the included eyes. That the rate of endophthalmitis after ICL implantation is very low was also shown by Allan et al. who investigated the rate of endophthalmitis between 1998 and 2006. Here, the rate was only 0.0167% in 17,954 ICL implantations^10^.

Concerning predictability, our results are comparable with those of Shimizu et al.^9^, Miao et al.^11^ and Packer et al.^12^. In our study, 89% of the eyes from the one-stage group and 86% from the two-stage group were within ± 0.50 D of the attempted SE correction. Target refraction within ± 1.0 D was one-stage 99% versus two-stage 99%. Regarding astigmatism correction, there were not significantly different results (≤ 0.5 D p = 0.41, ≤ 1.0 D). This is similar to the results of Reinstein et al.^13^ 74% of the eyes were target within ± 0.50 D of the attempted SE correction and 98% of the eyes target within ± 1.0 D.

Regarding stability, the one-stage group showed a lower myopic regression of 0.00 ± 0.40 D compared with the two-stage group of − 0.20 ± 0.40 D at the final control, but this is of no clinical relevance because of the small difference between the two groups. This may be because the follow-up in the two-stage group was slightly, but significantly longer by 2 months. Comparable results are described in the study by Igarashi et al.^14^ from 2022, in which a myopic regression of − 0.20 ± 0.43 D occurred at 2-year follow-up and in the study by Rateb et al.^15^, in which a myopic regression of -0.40 ± 0.2 D was observed after 1 year.

Concerning vaulting, the one-stage group showed higher values than the two-stage group. However, this could be due to the change of the ICL model in the one-stage group from V4c (18 eyes) to the latest model V5 (82 eyes) in the one-stage group, which might be a bias in this study. In the two-stage group, 42 eyes had the V4c model and only 36 eyes the V5 model. As the V5 features a larger optic zone, a higher vault as compared to V4c might result. Even if total lens diameter between V4c and V5 might be similar, the larger optic zone might lead to increased rigidity or a more convex shape.

Low vault values are the biggest risk factor for the development of secondary cataract. In our study, there was no correlation between low vault and cataract development. This is also been shown in studies from Nakamura et al.^5^ or Moya et al.^6^. The reduction in vault can be explained by many factors, like pupil movement, anterior chamber depth, horizontal corneal diameter, sulcus-to-sulcus-diameter^16^, age-related increase in the thickness of the crystalline lens as well as the fixed position of the ICL haptics^8^. Longer follow-up is necessary to evaluate whether the changes in vaulting will have any impact on cataract formation in the long term.

Comparison of endothelial cell number loss showed less loss in the one-stage group compared with the two-stage group (1.3% vs. 4.3%). As both the V4c and the V5 model share the 360 µm central aquaport, differences in endothelial cell count might be more attributable to surgical technique than to the ICL model. Rateb et al.^15^ reported an endothelial cell number loss of 3.3% by a 12-month follow-up. Zhang et al.^17^ reported that the lens type plays a significant role in the development of endothelial cell number loss postoperatively. He compared the V4 and V4c models, whose significant difference is the aquaport in the V4c model, which makes aqueous circulation more physiological. While an effect on endothelial cells is expected here, only models already with an aquaport were included in this study. Since the only difference between V4c and V5 is a slightly larger optic of V5, no clear explanation for the difference in endothelial cell number can be given. Many authors claim that the endothelial cell loss can be explained mainly by the surgery itself and should not be interpreted as a long-term effect. Overall, it should be emphasized that the loss of 1–4% is within the expected range and thus clinically comparable. Since a difference can already be seen one day postoperatively, no further loss is expected over time, resulting in little risk for corneal decompensation.

Regarding IOP, values remained stable through the entire follow-up. In the one-stage group, there were 6 eyes (6%) one day postoperative with a mild transient increase in IOP > 21 mmHg, in the two-stage group 2 eyes (2.6%). At the last follow up, 2 eyes (2%) in the one-stage group and no eye (0%) in the two-stage group had an increased IOP > 21 mmHg. This had no clinical relevance in all cases and was treated with pressure-lowering eye drops in the short term if necessary. The pressure-lowering eye drops brimonidin and bimatoprost were used as a combined therapy only temporarily for a maximum of 4 weeks and not long-term. None of these cases had an increased risk of glaucoma. This is similar to the results of Montes-Mico et al.^18^ and of Zhang et al.^17^.

In both groups halos were the most frequently perceived postoperative symptoms (one-stage 4% vs. two-stage 5.1%), which normalized over time in all patients and did not cause disturbing subjective complaints. This is also reported by Mohr et al.^19^; 90% of the patients perceived halos after surgery, but only 10% of the patients felt disturbed by them^19^. An equally high percentage of 84.3% was described in the study by Aruma et al.^20^. This seems to be relatively high compared to the results of this study, but Mohr et al.^19^ and Tana-Rivero et al.^21^ have shown that the perception of halos increases after the age of 40. In our study, the mean age in the one-stage group was 33.4 ± 6.5 years and in the two-stage group 33.1 ± 6.8 years, so the percentage of perception of halos is correspondingly lower. None of the patients developed a cataract during the follow-up period, but the relatively short follow-up period of 1.1 ± 0.8 years in the one-stage group and 1.3 ± 0.5 years in the two-stage group must be considered. Kocova et al.^22^ reported the incidence of cataract development after ICL implantation at a follow-up of 15 years, Chen et al.^16^ at a follow-up of 5 years. In both groups a similar percentage of patients developed macular edema postoperatively (one-stage 4% vs. two-stage 3.8%), which was treated in all cases without complications and caused no further problems. As described by Zhang et al. possible mechanisms of macular edema include rubbing of the ICL against the posterior iris surface, detachment of the posterior vitreous, or retinal traction by the vitreous^17^. To date, there have been few reports of postoperative macular edema after ICL implantation; however, it has been reported that postoperative macular edema can develop even in phakic eyes with an intact posterior capsule, but it is usually self-limiting^23^. This is further confirmed by the fact that all macular edema (4% in the one-stage group vs. 3.8% in the two-stage group) in our study regressed under conservative management with topical drop therapy alone. In both groups one eye experienced ICL rotation postoperatively (in the one-stage group because of high vault and in the two-stage group because of a rest-astigmatism of 1.25 D) but in all cases we waited and did not re-operate due to lack of symptoms. This was due to the low rotation and the lack of subjective complaints of the patients. That realignment or exchange is rarely performed after ICL implantation is also reported by Wei et al.^24^.

Possible confounding factors and limitations to this study include the retrospective design of this study and the gender distribution with more female patients in the two-stage vs. the one stage group (87 vs. 64%). Nevertheless, gender has not yet been shown to have an impact on the intra- or postoperative outcome in ICL implantation. Moreover, our study design involved simultaneous preoperative planning for both eyes, which differs from a truly sequential approach where outcomes in the first eye influence lens calculation and surgical planning in the second eye. Additionally, studies with follow-up beyond year one reported in this study are needed to better compare safety and efficacy at 2, 5 and 10 years. Finally, sample size was calculated according to a non-inferiority in efficacy. To show non-inferiority concerning rare adverse events like endophthalmitis, sample size would have needed to be considerably larger. These confounders must be taken into consideration when interpreting the results of this retrospective non-interventional study.

In summary, this study demonstrates that both one-stage simultaneous bilateral ICL implantation and two-stage delayed bilateral ICL implantation are safe and effective surgical options for the correction if high myopia and myopic astigmatism. Our data might serve as a basis for a prospective observational study in order to gain more robust evidence about the safety of a one-stage procedure.

## Methods

The database of the University Eye Hospital Munich, Ludwig Maximilians-University and Smile Eyes Linz, Austria were screened for eyes that had undergone ICL implantation between 2013 and 2020. Eyes were subdivided in two groups to compare one-stage same-day surgery versus two-stage surgery at an interval of 1 day (17 patients), 2 days (19 patients) or 1 week (2 patients). Eyes were eligible for inclusion in this study if a minimum follow-up of 12 months was met. The efficacy index (UDVA postOP/CDVA preOP) at last follow-up was defined as primary end point. Variables analyzed were preoperative, 1-day and last follow-up uncorrected distance (UDVA) and corrected distance visual acuity (CDVA), manifest refraction, refractive spherical equivalent (SEQ), astigmatism, age, endothelial cell count (ECD), intraocular pressure (IOP) and ICL vaulting. In addition to the primary end point defining efficacy, safety was compared by analyzing the documented unexpected outcomes (e.g., ocular pressure, location of the ICL).

This study was approved by the ethics committee of the medical faculty of the Ludwig-Maximilians-University of Munich (project number: 22-0810; November 14th, 2022). All study procedures were performed in accordance with the principles of the Declaration of Helsinki and all patients provided written informed consent.

### ICL implantation

According to the current guidelines of the German Commission on Refractive Surgery (KRC) for evaluation and quality assurance as of June 2022, ICL implantation was performed for myopia from − 1.0 D and hyperopia from + 1.0 D as well as astigmatism. Prerequisites were age > 18 years, an endothelial cell density of at least 2.000/mm^2^ and an anterior chamber depth of at least 2.8 mm for myopia (or 3.0 mm for hyperopia, not performed in this study). In addition, it is recommended that follow-up corneal endothelial cell density testing be performed at least annually for all phakic IOLs.

All ICL implantations were performed by the same highly experienced surgeon (S.P.) as described previously^19^. There was a switch from lens type V4c (18 eyes) to the latest model V5 (82 eyes) in the one-stage group and in the two-stage group 42 eyes became the V4c model and 36 eyes the V5 model. In brief, lens sizing was based on preoperative measurements obtained with the IOL Master 700 (Carl Zeiss Meditec AG, Jena, Germany) and the Pentacam HR (Oculus Optikgeräte GmbH, Wetzlar, Germany). ICL power and sizing were calculated using the manufacturer’s online tool “STAAR surgical online calculation & ordering system”. Implantation was performed with a temporal 2.8 mm clear cornea incision. For toric ICLs, corneal marks indicating the required axis were performed by the surgeon at the slit lamp preoperatively. Intraoperatively, unpreserved lidocaine 1% was used for analgesia and limited pupil dilation. Eyefill HD (Bausch & Lomb, Rochester, NY, USA) was used as viscoelastic agent. After implantation, miosis was achieved by the intracameral injection of Miochol-E (Bausch & Lomb). Postoperatively, patients received dexamethasone 0.1% and tobramycin 0.3% eye drops (Tobradex®, Novartis, Basel, Switzerland) six times daily for one week and ketorolac 0.5% eye drops (Acular®, Allergan plc, Dublin, Ireland, and Abbvie, North Chicago, Illinois, USA) three times a day for 4 weeks. Additional preservative-free lubricants were recommended as needed.

### Refraction and visual acuity

Subjective refraction was determined using a phoropter and the standardized Jackson cross-cylinder method. Uncorrected distance visual acuity (UDVA) and corrected distance visual acuity (CDVA) were measured at a distance of 4 m.

### Vaulting

The ICL vaulting (= distance between crystalline lens and ICL) was determined from each of the manual 180-degree meridian Scheimpflug images (Pentacam HR, Oculus Optikgeräte GmbH, Wetzlar, Germany).

### Endothelial cell count

Endothelial cell count was measured by high-precision endothelial microscopy (CEM − 530; NIDEK, Gamagori, Japan) preoperatively, 1 day postoperatively and at the final follow-up.

### Statistical analysis

For comparison of the two groups, all statistical analyses were performed using SPSS Statistics for Mac (IBM SPSS Statistics 28; IBM, Armonk, NY, USA) and Microsoft Excel for Mac (version 16.65, 22,091,101; Microsoft, Redmond, USA). The significance level was set at p < 0.05. All data are presented as mean and standard deviation.

For sample size calculation, the efficacy index at final follow-up was defined as primary end point. Assuming a power of 80% (1–β), an Type I error rate (α) of 5%, an expected standard deviation of 0.2 according to a review of 27 efficacy studies by Packer et al.^25^ and a non-inferiority limit of 0.075, a sample size of 88 eyes per group (176 total) was calculated.

The hypotheses presented in this paper were tested using the following procedures: To calculate mean differences between two groups (e.g. gender, age), the t-test for independent samples was used. The applicable prerequisites such as interval scale level, normal distribution of the data or a group size with N > 30 and homogeneity of variances were checked before application. The Mann–Whitney *U* test was used as a nonparametric test to calculate differences between groups. Differences within a group were calculated after checking the normal distribution either with the *t* test for dependent samples or with the non-parametric Wilcoxon signed-ranks test.

## Data Availability

The datasets used and analyzed during the current study will be made available from the corresponding author on reasonable request.
